# Supplementary material for: Subthalamic Nucleus Deep Brain Stimulation Treats Parkinson’s Disease Patients with Cardiovascular Disease Comorbidity: A Retrospective Study of a Single Center Experience
Source: Brain Sci. 2022 Dec 29;13(1):70. doi: 10.3390/brainsci13010070 (PMC9857054; doi:10.3390/brainsci13010070)
Supplement: Supplementary file 1 [file brainsci-13-00070-s001.zip › brainsci-2087858-supplementary.pdf]

Table S1. Electrocardiogram, Echocardiography.

ASA classification and RCRI results

Case	ASA classification	RCRI index	LVPWT (mm)	EF%	Heart rate (bpm)	RV5+SV1 (mV)	ECG
1	3	0	11	70	97	1.86	normal ECG
2	2	0	8	75	78	2.74	normal ECG
3	3	0	8	71	73	1.97	normal ECG
4	3	0	9	73	76	1.08	Sinus heart rate, abnormal Q waves
5	3	0	9	67	85	2.35	normal ECG
6	3	1	9	73	78	1.5	Sinus heart rate, abnormal Q waves
7	3	1	9	64	102	2.39	sinus tachycardia with atrial premature beat bigemine and mild ST-T changes
8	3	0	10	74	88	1.48	normal ECG
9	3	0	9	74	69	3.04	normal ECG
10	3	0	9	67	105	3.37	Sinus tachycardia with mild ST changes
11	3	0	9	73	68	2.2	normal ECG
12	3	1	9	66	88	2	normal ECG
13	3	0	10	76	68	2.53	normal ECG
14	3	0	9	74	71	2.72	normal ECG
15	3	1	10	70	72	2.34	normal ECG
16	3	0	10	62	71	2.88	Sinus heart rate with atrial premature beats and mild ST-T changes
17	3	1	9	76	55	2.94	sinus bradycardia
18	3	0	9	73	87	3.16	normal ECG
19	3	1	9	66	56	2.54	sinus bradycardia
20	3	0	11	67	60	1.8	Atrial pacing rhythm, prolonged AV conduction, incomplete right bundle branch block, and left anterior fascicular block.
21	3	0	9	73	85	2.07	Sinus heart rate with abnormal Q waves
22	3	1	9	61	69	3.47	Sinus bradycardia, premature ventricular contractions
23	3	0	7	64	71	1.96	Sinus rhythm, complete right bundle branch block
24	3	0	9	63	102	2.14	sinus tachycardia
25	3	1	9	58	93	3.21	Atrial fibrillation with mild T-wave changes
26	3	2	8	75	74	2.62	normal ECG

27	3	1	10	71	83	3.34	normal ECG
28	3	1	9	73	71	2.72	normal ECG
29	3	1	9	67	63	2.81	normal ECG
30	3	2	8	73	85	2.27	normal ECG
31	3	2	10	70	78	2.74	sinus bradycardia
32	3	3	10	61	91	3.05	Sinus heart rate, abnormal Q waves
33	3	2	9	72	69	2.99	normal ECG
34	3	1	8	76	76	2.82	normal ECG
35	3	2	10	72	68	3.01	sinus bradycardia
36	3	2	10	63	89	2.40	normal ECG
37	3	1	9	72	88	2.52	sinus bradycardia
38	3	1	/	/	82	/	/
Average	2.97±0.16	0.76±0.82	9.16 ± 0.83	69.59 ± 4.96	78.53 ± 12.41	2.51 ± 0.57	

Numbers indicate means ± standard deviations (SD). ASA: American Society of Anesthesiologists, RCRI: Revised Cardiac Risk Index , LVPWT :Left Ventricular Posterior Wall Thickness, EF: ejection fraction. bpm, beats per minute. ECG: Electrocardiograph ,RV5: R-wave in lead V5，SV1: S-wave in lead V1.AV: atrioventricular .“/ ” means data lost.
